# Associations of Chinese visceral adiposity index and new-onset stroke in middle-aged and older Chinese adults: an observational study

**DOI:** 10.1186/s12944-023-01843-x

**Published:** 2023-06-19

**Authors:** Hongyu Zhang, Qi Zhan, Fayan Dong, Xueting Gao, Fanyue Zeng, Jiahao Yao, Yifan Gan, Shuhuai Zou, Jianheng Gu, Hongqian Fu, Xuefeng Wang

**Affiliations:** Department of Neurosurgery, The Fourth Affiliated Hospital of Harbin Medical University, Harbin, 150001 China

**Keywords:** Chinese visceral adiposity index (CVAI), New-onset stroke, Prospective cohort study, Middle-aged and older Chinese, China Health and Retirement Longitudinal Study (CHARLS)

## Abstract

**Background:**

Stroke represents the second most prevalent contributor to global mortality. The Chinese Visceral Adiposity Index (CVAI) serves as an established metric for assessing visceral adiposity in the Chinese population, exhibiting prognostic capabilities. This investigation aimed to explore the association of CVAI and new-onset stroke among middle-aged and older Chinese populations.

**Methods:**

The study employed data from the 2011 and 2018 China Health and Retirement Longitudinal Study (CHARLS) to assess the association of CVAI and the incidence of new-onset stroke. Utilizing a directed acyclic graph (DAG), 10 potential confounders were identified. Moreover, to explore the association between CVAI and new-onset stroke, three multifactor logistic regression models were constructed, accounting for the identified confounders and mitigating their influence on the findings.

**Results:**

The study comprised 7070 participants, among whom 417 (5.9%) experienced new-onset strokes. After controlling for confounding variables, regression analysis suggested that the new-onset stroke’s highest risk was linked to the fourth quartile (Q4) of the CVAI, with an odds ratio (OR) of 2.33 and a 95% confidence interval (CI) of 1.67–3.28. The decision tree analysis demonstrated a heightened probability of new-onset stroke among hypertensive individuals with a CVAI equal to or greater than 83, coupled with a C-reactive protein level no less than 1.1 mg/l. Age seemed to have a moderating influence on the CVAI and new-onset stroke association, exhibiting a more prominent interaction effect in participants under 60 years.

**Conclusions:**

In middle-aged and older Chinese populations, a linear relationship was discerned between CVAI and the probability of new-onset stroke. CVAI provides a predictive framework for stroke incidence in this demographic, laying the groundwork for more sophisticated risk prediction models that improve the precision and specificity of stroke risk evaluations.

## Background

Stroke is one of the most prevalent cerebrovascular disease, encompassing both ischemic and hemorrhagic types. Stroke, arising from organic brain injuries, is defined by its abrupt onset and the quick emergence of either localized or diffuse brain function impairments. Globally, stroke ranks as the second most common cause of mortality and a principal factor in morbidity [1]. The United States and Japan report estimated annual incidences of approximately 795,000 and 191,000 new-onset and recurrent strokes, respectively [2]. In China, approximately two million new-onset stroke cases occur annually, exhibiting an 8.7% growth rate [3]. Although age-standardized mortality and Disability-Adjusted Life Year (DALY) rates for stroke have declined, the global burden of disease (GBD) estimates reveal an increase in overall incidence and crude stroke burden rates between 1990 and 2019 [4]. Consequently, stroke presents a significant public health challenge and imposes a considerable financial strain on individuals and society. Prior studies have identified hypertension, diabetes, atrial fibrillation, dyslipidemia, visceral obesity, smoking, and advanced age as factors associated with an elevated risk of new-onset stroke [5–8]. Proactive management and mitigation of these risk factors can substantially reduce stroke incidence.

Visceral obesity represents a global health issue, with World Health Organization data indicating that over 40% of adults worldwide are overweight, while 13% are classified as obese [9, 10]. China is home to an estimated 89.6 million obese individuals [11], constituting the largest obese population globally. Standard indices employed to evaluate adipose tissue encompass hip girth, adipose tissue proportion, and the waist-height ratio [12]. However, these indices independently fail to provide a comprehensive assessment of visceral adipose distribution. Xia et al. [13] developed the Chinese visceral adiposity index (CVAI) based on the visceral adiposity index (VAI) and incorporated body mass index (BMI), age, high-density lipoprotein cholesterol (HDL-C), triglycerides (TG), and Waist circumference (WC) to better reflect visceral fat characteristics in the Chinese population. Existing research [14] suggests that CVAI functions as a potent and credible measure of visceral adipose tissue dysregulation.

In recent years, the prognostic utility of CVAI has gained increasing interest in numerous studies. Xie et al. [15] proposed that CVAI might function as a substantial prognosticator of cardiometabolic risk in adults experiencing growth hormone deficiency, facilitating the recognition of potential cardiovascular ailments and vascular occurrences. Han et al. [16] documented a positive correlation between CVAI and the susceptibility to type 2 diabetes mellitus, while Chen et al. [17] posited that CVAI might assist in diagnosing non-alcoholic fatty liver disease. However, limited research has examined the direct relationship between CVAI and new-onset stroke. Although previous investigations [18, 19] have explored the association between VAI and stroke incidence, VAI calculations were based on equations established for Caucasian populations. CVAI, due to considerable differences in body fat distribution across ethnic groups [20], is better suited for Chinese populations. CVAI is posited to influence the onset of cerebrovascular diseases, like stroke, potentially through mechanisms such as dyslipidemia and insulin resistance. Given that inflammation and thrombosis are key players in stroke pathogenesis, we hypothesize that higher CVAI may affect the incidence of new strokes. Consequently, as a China-specific metabolic marker, CVAI holds significance in predicting new-onset stroke.

Drawing upon the publicly available China Health and Retirement Longitudinal Study (CHARLS) database [21], this investigation delved into the association between CVAI and new-onset stroke among middle-aged and older Chinese individuals, in addition to assessing the predictive utility of CVAI for stroke incidence.

## Methodological approaches and materials

### Investigated cohort

Data for this cohort investigation were procured from CHARLS (http://charls.pku.edu.cn/). The study utilized baseline information gathered in 2011 and follow-up data from 2018. CHARLS, an open-access resource, is a continuous, nationally representative cohort study encompassing participants aged 45 years and above from over 400 villages and communities within 25 provinces, districts, and cities. The database compiles a plethora of information, including personal, familial, financial, health, employment, and neighborhood data. The primary objective is to amass high-quality microdata that typifies middle-aged and older Chinese households, thereby assessing the nation’s aging population. The dataset from CHARLS categorizes individuals aged 45 years and beyond as middle-aged and elderly, a classification that aligns with the United Nations’ definition of older adults as being 60 years and above [22–25], in line with previous studies [26]. This investigation strictly adhered to relevant CHARLS guidelines and recommendations.

**Fig. 1:**
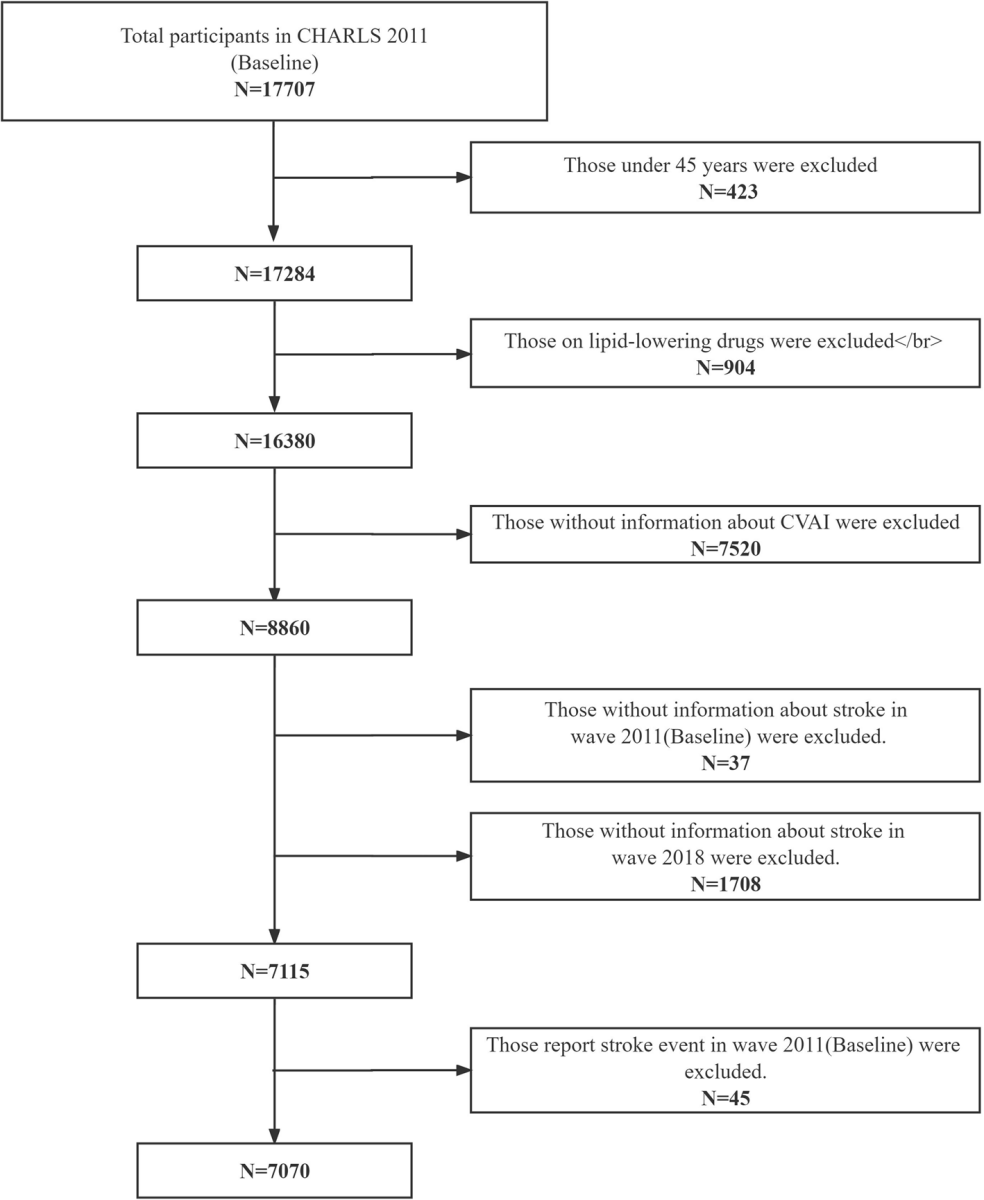
Flow diagram illustrating participant selection from the CHARLS cohort. CHARLS, China Health and Retirement Longitudinal Study

Prior to the survey, participants willingly signed informed consent forms. Of the 17,707 respondents in the 2011 baseline survey, each provided fasting blood samples, underwent baseline physical examinations, and completed questionnaires. Subsequently, 10,683 participants were excluded based on the following criteria: age under 45 years (*n* = 423), lipid-lowering drug usage history (*n* = 904), incomplete CVAI data (*n* = 7520), unavailable stroke information in the 2011 wave (*n* = 23), unavailable stroke information in the 2018 wave (*n* = 1708), and stroke presence at baseline (*n* = 105) (Fig. 1).

### Measurement

#### Assessment of the CVAI

The CVAI amalgamates anthropometric and functional parameters, providing insight into visceral adipose tissue distribution. CVAI computation employed the subsequent formula [16]:

$${\varvec{M}}{\varvec{e}}{\varvec{n}}:{\varvec{C}}{\varvec{V}}{\varvec{A}}{\varvec{I}}=-267.93+0.68\times {\varvec{a}}{\varvec{g}}{\varvec{e}}\boldsymbol{ }({\varvec{y}})+0.03\times {\varvec{B}}{\varvec{M}}{\varvec{I}}({\varvec{k}}{\varvec{g}}/{{\varvec{m}}}^{2})+4.00\times {\varvec{W}}{\varvec{C}}({\varvec{c}}{\varvec{m}})+22.00\times {\varvec{l}}{\varvec{g}}{\varvec{T}}{\varvec{G}}({\varvec{m}}{\varvec{m}}{\varvec{o}}{\varvec{l}}/{\varvec{L}})-16.32\times {\varvec{H}}{\varvec{D}}{\varvec{L}}-{\varvec{C}}({\varvec{m}}{\varvec{m}}{\varvec{o}}{\varvec{l}}/{\varvec{L}})$$

$${\varvec{W}}{\varvec{o}}{\varvec{m}}{\varvec{e}}{\varvec{n}}:\boldsymbol{ }{\varvec{C}}{\varvec{V}}{\varvec{A}}{\varvec{I}}\boldsymbol{ }=\boldsymbol{ }-187.32\boldsymbol{ }+\boldsymbol{ }1.71\boldsymbol{ }\times \boldsymbol{ }{\varvec{a}}{\varvec{g}}{\varvec{e}}\boldsymbol{ }({\varvec{y}})+\boldsymbol{ }4.23\boldsymbol{ }\times \boldsymbol{ }{\varvec{B}}{\varvec{M}}{\varvec{I}}\boldsymbol{ }({\varvec{k}}{\varvec{g}}/{{\varvec{m}}}^{2})\boldsymbol{ }+\boldsymbol{ }1.12\boldsymbol{ }\times \boldsymbol{ }{\varvec{W}}{\varvec{C}}\boldsymbol{ }({\varvec{c}}{\varvec{m}})\boldsymbol{ }+\boldsymbol{ }39.76\boldsymbol{ }\times \boldsymbol{ }{\varvec{l}}{\varvec{g}}\boldsymbol{ }{\varvec{T}}{\varvec{G}}\boldsymbol{ }({\varvec{m}}{\varvec{m}}{\varvec{o}}{\varvec{l}}/{\varvec{L}})\boldsymbol{ }-11.66\boldsymbol{ }\times \boldsymbol{ }{\varvec{H}}{\varvec{D}}{\varvec{L}}-{\varvec{C}}\boldsymbol{ }({\varvec{m}}{\varvec{m}}{\varvec{o}}{\varvec{l}}/{\varvec{L}}).$$

#### Evaluation of new-onset strokes

Incidence of new-onset stroke was evaluated by inquiring respondents, “Have you ever been diagnosed with a stroke by a doctor?” and “When did you first discover the condition?” If an affirmative response was provided during the follow-up, and the participant had not reported a stroke in the baseline survey but did so during the follow-up, they were deemed to have experienced a new-onset stroke [27].

#### Assessment of potential confounders

Covariates considered in the directed acyclic graph (DAG) encompassed age, sex, marital status, education, location, smoking, drinking, low-density lipoprotein cholesterol (LDL-C), BMI, WC, diabetes, hypertension, HDL-C, TG, and C-reactive protein (CRP). These covariates were classified into socio-demographic characteristics, health-related behaviors, and anthropometric measurements. Socio-demographic attributes were classified into five categories: age, sex, educational attainment (primary school or below, middle school, and high school or above), residential location (urban and rural), and marital status (married and unpartnered). Health-related behaviors encompassed four classifications: smoking status (non-smoker, former smoker, and current smoker), as well as alcohol consumption habits (none, less than once a month, and more than once a month). Anthropometric measurements, such as TG, BMI, HDL-C, CRP, LDL-C, and WC, were obtained using standardized techniques [21, 28]. Waist circumference was measured utilizing a specific protocol with the following steps: First, we utilized a non-elastic tape measure as our equipment. Participants were instructed to stand upright, their feet positioned shoulder-width apart, and arms relaxed, perpendicular to the body. The waist circumference was gauged using the tape measure without clothing, at the level of the navel. While the participant was breathing calmly, the investigator fastened the measuring tape snugly around the body without compressing the skin and read the measurement to two decimal places at the end of the participant’s exhalation. This process was repeated thrice, and the average of these three measurements was recorded as the final result. All measurement instruments were appropriately calibrated to guarantee an error margin of less than 0.5 cm in waist circumference measurements. Comprehensive methodologies are accessible on the CHARLS website (http://charls.pku.edu.cn/).

**Fig. 2:**
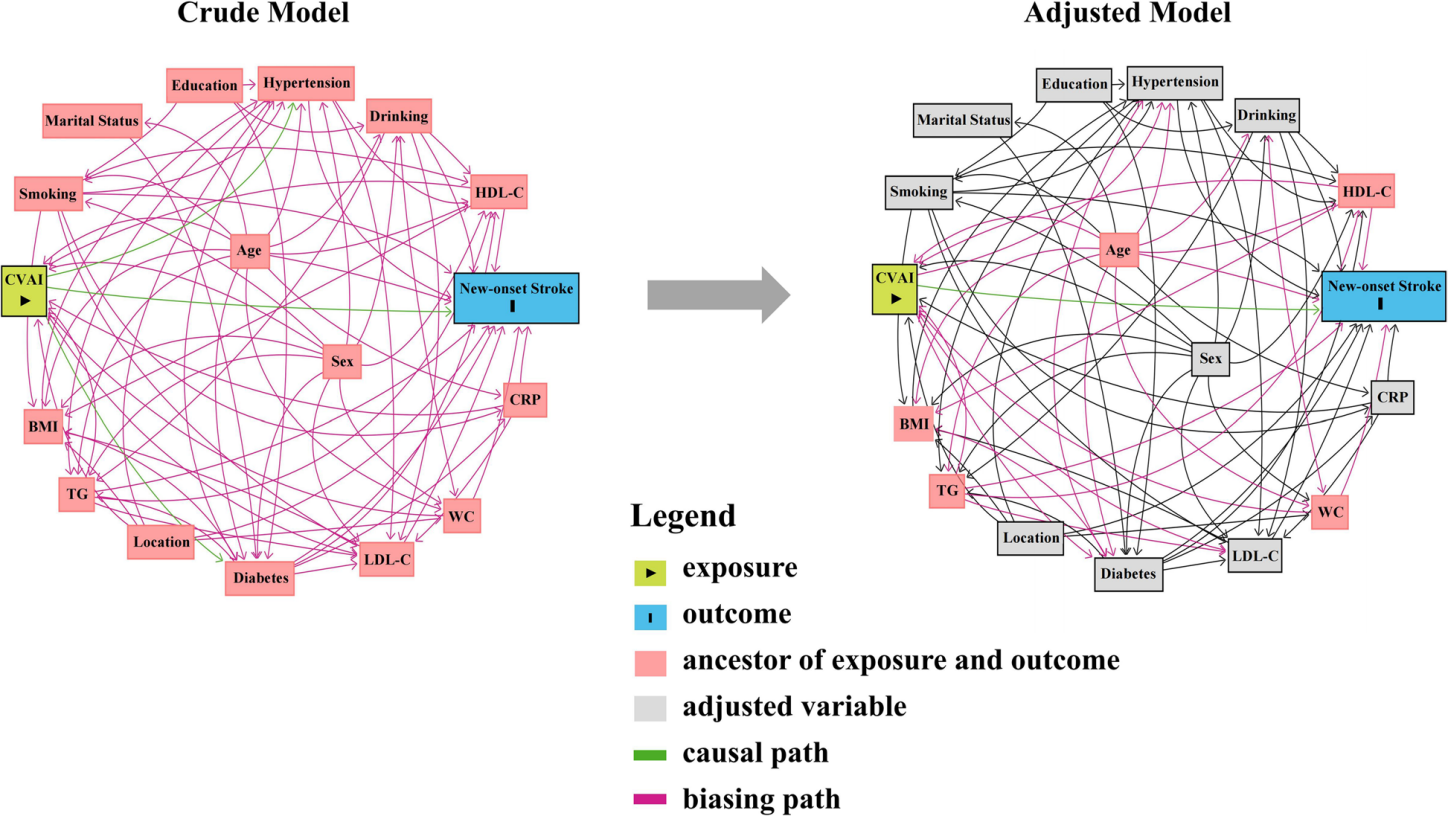
DAG illustrating the relationship between CVAI and new-onset stroke. DAG displays potential confounding pathways in both the crude model (left) and the adjusted model (right). DAG, directed acyclic graph; CVAI, Chinese Visceral Adiposity Index

In this study, a DAG [29, 30] was employed to select a minimally sufficient set of confounding variables (Fig. 2). DAG execution was facilitated by dagitty.net [31]. Given that age, WC, BMI, TG, and HDL-C were already incorporated in the CVAI equation, they were not adjusted for in the regression model. Through the DAG, relevant adjustment factors and eliminated confounding variables were identified, thereby minimizing bias in the final model’s estimated association between CVAI and new-onset stroke. Adjusted variables encompassed sex, marital status, education, location, smoking, drinking, diabetes, hypertension, LDL-C, and CRP levels.

### Statistical analysis

For non-normally distributed data, medians and quartiles (Q1–Q3) were computed, while categorical variables were expressed as proportions. Baseline characteristics and CVAI were stratified according to the presence or absence of new-onset stroke. Data comparisons were conducted using the t-test, Mann–Whitney U, and Chi-square test, as appropriate. In order to evaluate the odds ratio (OR) and 95% confidence interval (CI) of new-onset stroke in relation to CVAI as continuous (per interquartile range [IQR] increment) or categorical (quartiles) variables, three logistic regression models were implemented: Model 1 depicted crude associations between CVAI and new-onset stroke; Model 2 incorporated further adjustments for sex, educational attainment, residential location, and marital status; Model 3 encompassed adjustments from Model 2, alongside health behavior and disease history factors, such as smoking status, alcohol consumption habits, LDL-C, CRP, and hypertension and diabetes histories.

A restricted cubic splines (RCS) analysis incorporating three knots at the 10^th^, 50^th^, and 90^th^ percentiles of CVAI was undertaken to investigate potential linear associations and illustrate dose–response relationships between CVAI and new-onset stroke. Interaction analyses were carried out independently to evaluate moderating influences among socio-demographic characteristics and health-related behaviors in the connection between CVAI and new-onset stroke. Models integrated multiplicative interaction terms, and likelihood-ratio tests were conducted to appraise the interaction. Furthermore, a decision tree was utilized to examine the predictive merit of CVAI.

All statistical evaluations were performed utilizing R 4.1. The ‘ANOVA’ function within the rms R package enabled RCS analysis. The decision tree construction employed the ‘rpart’ package. A two-tailed *P*-value less than 0.05 denoted statistical significance in the analysis.

## Results

### Baseline attributes

Table 1 presents participant characteristics. The study encompassed a total of 7,070 individuals (median age: 58 years; 3,158 males: 45%; 3,912 females: 55%), among which 417 (5.9%) experienced new-onset strokes, while the rest remained healthy. The baseline median (IQR) CVAI for participants was 93.9 (64.1–124.4). Notably, CVAI was substantially elevated in individuals with new-onset stroke compared to healthy counterparts. Distinct characteristics were observed among participants with new-onset stroke; they exhibited a higher likelihood of being older, residing unaccompanied, being former smokers, having hypertension and diabetes, while exhibiting higher BMI, WC, TG, CRP, and LDL-C levels, and displaying lower HDL-C levels (*P* < 0.05).

**Table 1:** Characteristics of study participants categorized by a new-onset stroke occurrence at baseline (*N* = 7070)

	Total (*n* = 7070)	Non-Stroke (*n* = 6653)	Stroke (*n* = 417)	*P*
Age, n (%)				< 0.010
45–60	4106 (58.1)	3914 (58.8)	192 (46.0)	
> 60	2964 (41.9)	2739 (41.2)	225 (54.0)	
Sex, n (%)				0.530
Male	3158 (44.7)	2965 (44.6)	193 (46.3)	
Female	3912 (55.3)	3688 (55.4)	224 (53.7)	
Marital Status, n (%)				0.010
Married	6310 (89.3)	5954 (89.5)	356 (85.4)	
Alone	760 (10.8)	699 (10.5)	61 (14.6)	
Education, n (%)				0.190
Primary school or below	4941 (69.9)	4634 (69.7)	307 (73.6)	
Middle school	1439 (20.4)	1368 (20.6)	71 (17.0)	
High school or above	690 (9.8)	651 (9.8)	39 (9.4)	
Location, n (%)				0.660
Village	451 (6.4)	427 (6.4)	24 (5.8)	
City	6619 (93.6)	6226 (93.6)	393 (94.2)	
**Smoking, n (%)**				0.010
Non-smoker	4416 (62.5)	4169 (62.7)	247 (59.2)	
Ex-smoker	526 (7.4)	480 (7.2)	46 (11.0)	
Current smoker	2128 (30.1)	2004 (30.1)	124 (29.7)	
Drinking, n (%)				0.970
None of these	4727 (66.9)	4448 (66.9)	279 (66.9)	
Drink but less than once a month	564 (8.0)	532 (8.0)	32 (7.7)	
Drink more than once a month	1779 (25.2)	1673 (25.2)	106 (25.4)	
BMI (kg/m^2^)	23.0 (20.9, 25.5)	23.0 (20.8, 25.5)	23.8 (21.6, 26.6)	< 0.010
WC (cm)	84.0 (77.8, 91.0)	84.0 (77.5, 91.0)	88.0 (80.6, 95.0)	< 0.010
Diabetes^a^, n (%)				< 0.010
No	6017 (85.1)	5686 (85.5)	331 (79.1)	
Yes	1053 (14.9)	967 (14.5)	86 (20.9)	
Hypertension^a^, n (%)				< 0.010
No	4443 (62.8)	4273 (64.2)	170 (40.8)	
Yes	2627 (37.2)	2380 (35.8)	247 (59.2)	
TG (mg/dl)	103.5 (74.3, 146.0)	102.7 (73.5, 146.0)	113.3 (85.9, 153.1)	< 0.010
HDL-C (mg/dl)	49.8 (41.0, 60.3)	49.9 (41.0, 60.3)	47.2 (39.4, 57.2)	< 0.010
LDL-C (mg/dl)	113.7 (93.6, 135.3)	113.7 (93.6, 135.3)	117.1 (95.1,139.6)	0.020
CRP (mg/l)	0.9 (0.5, 1.9)	0.9 (0.5, 1.8)	1.2 (0.6, 2.2)	< 0.010
CVAI	93.9 (64.1, 124.4)	92.8 (62.8, 123.2)	112.7 (83.6, 141.3)	< 0.010
Quartiles of CVAI, n (%)				< 0.010
Q1	1768 (25.0)	1710 (25.7)	58 (13.9)	
Q2	1767 (25.0)	1681 (25.3)	86 (20.6)	
Q3	1767 (25.0)	1656 (24.9)	111 (26.6)	
Q4	1768 (25.0)	1606 (24.1)	162 (38.9)	

*Abbreviations*: *BMI* Body mass index, *WC* Waist circumference, *TG* Triglyceride, *HDL-C* High-density lipoprotein cholesterol, *LDL-C* Low-density lipoprotein cholesterol, *CRP* C-reactive protein, *CVAI* Chinese visceral adiposity index

^a^Yes represents participants with hypertension or diabetes, while No indicates participants without hypertension or diabetes. In the CHARLS database, hypertension was defined by any of the following criteria: self-reported hypertension, a diastolic blood pressure of 90 mmHg or higher, or a systolic blood pressure of 140 mmHg or greater. Diabetes was defined by either self-reported, physician-diagnosed diabetes, a fasting plasma glucose level of 126 mg/dl or more, or a glycated hemoglobin level of 6.5% or above

### Dose–response association of CVAI and new-onset stroke

**Fig. 3:**
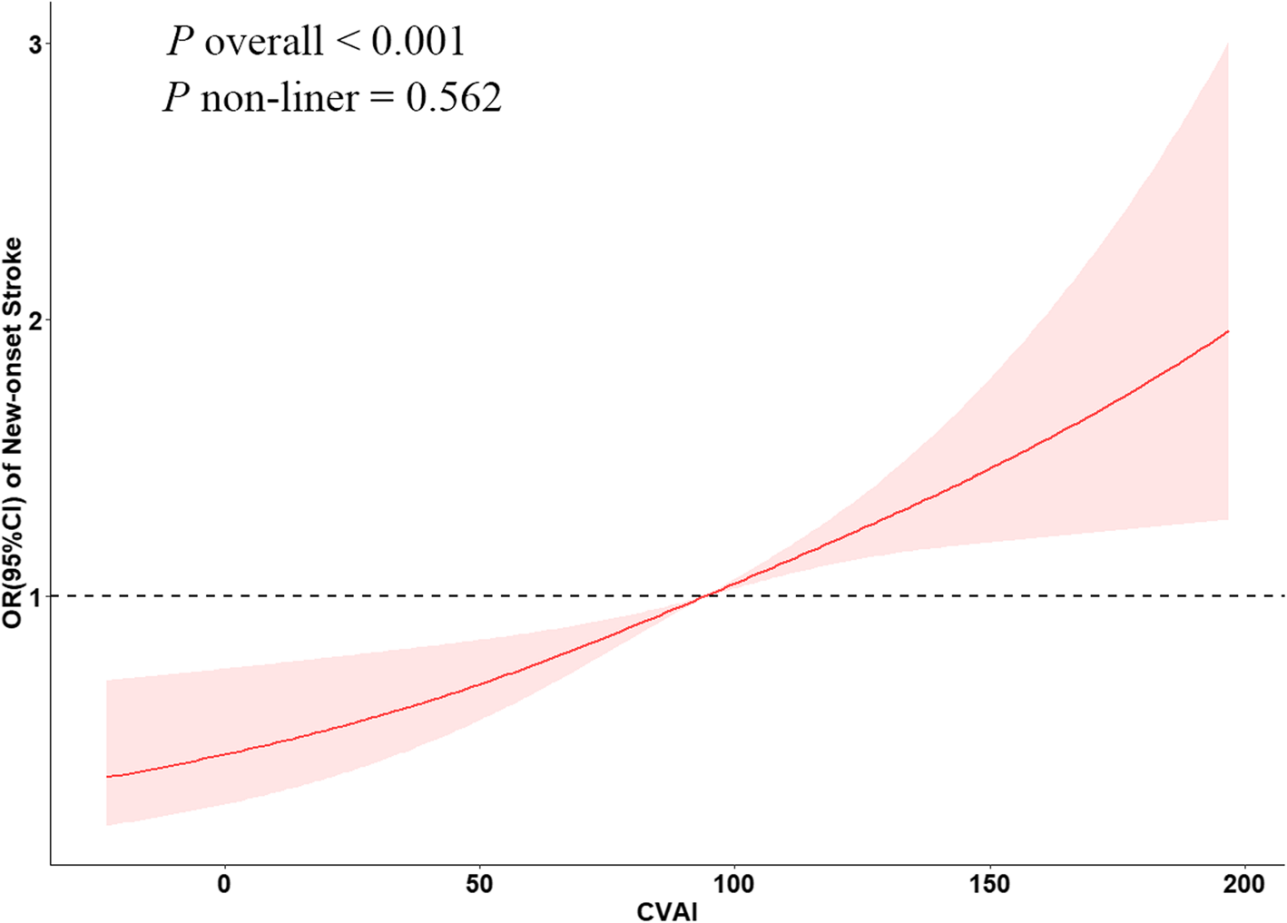
Association of CVAI and the risk of new-onset stroke utilizing the adjusted cubic spline model. The model accounts for confounding factors, including sex, educational background, geographical location, marital status, smoking habits, alcohol consumption, LDL-C, and CRP, as well as histories of hypertension and diabetes. The graphical representation demonstrates a linear association of CVAI and the hazard of new-onset stroke. CVAI, Chinese Visceral Adiposity Index; LDL-C, low-density lipoprotein cholesterol; CRP, C-reactive protein

Table 2 delineates the correlation between the incidence of novel stroke events and CVAI quartiles. The analysis revealed a significant escalation in stroke risk concomitant with elevated CVAI quartiles after accounting for confounding factors such as sex, marital status, LDL-C, smoking habits, CRP, hypertension history, geographical location, alcohol consumption, educational background, and diabetes prevalence (*P* for trend < 0.001, OR: 2.33, 95% CI: 1.67–3.28). Moreover, Q4 exhibited the most pronounced susceptibility to novel stroke occurrences in comparison to Q1. CVAI demonstrated a marked association with the emergence of new stroke cases. Upon assessment as a continuous variable, evidence suggested a 57% surge in stroke risk for each IQR increment in CVAI (OR: 1.57, 95% CI: 1.34–1.84). Figure 3 portrays the linear dose-response relationship between CVAI and novel stroke risk (*P*_overall_ < 0.001, *P*_nonlinear_ = 0.562). The investigation determined a progressive augmentation in stroke risk, concomitant with a consistent elevation in CVAI. The CVAI threshold of 94.1 emerged as a determinant of novel stroke events.

**Table 2:** Association of CVAI and new-onset stroke in the CHARLS cohort

	Model 1	*P*	Model 2	*P*	Model 3	*P*
CVAI per IQR	1.83 (1.58, 2.11)	< 0.001	1.91 (1.66, 2.21)	< 0.001	1.57 (1.34, 1.83)	< 0.001
Q1	ref		ref		ref	
Q2	1.51 (1.08, 2.13)	0.018	1.66 (1.18, 2.36)	0.004	1.51 (1.07, 2.15)	0.021
Q3	1.96 (1.43, 2.75)	< 0.001	2.25 (1.62, 3.17)	< 0.001	1.83 (1.30, 2.59)	0.001
Q4	2.98 (2.20, 4.07)	< 0.001	3.37 (2.46, 4.66)	< 0.001	2.33 (1.67, 3.28)	< 0.001
*P* for trend	< 0.001		< 0.001		< 0.001	

Model 1: Unadjusted model

Model 2: Incorporating adjustments for sex, education level, location, and marital status

Model 3: Subsequently incorporating additional factors from Model 2, along with health behavior and medical history, encompassing smoking status, alcohol consumption patterns, LDL-C, CRP, history of hypertension and diabetes

### Decision tree analysis of CVAI for new-onset stroke

**Fig. 4:**
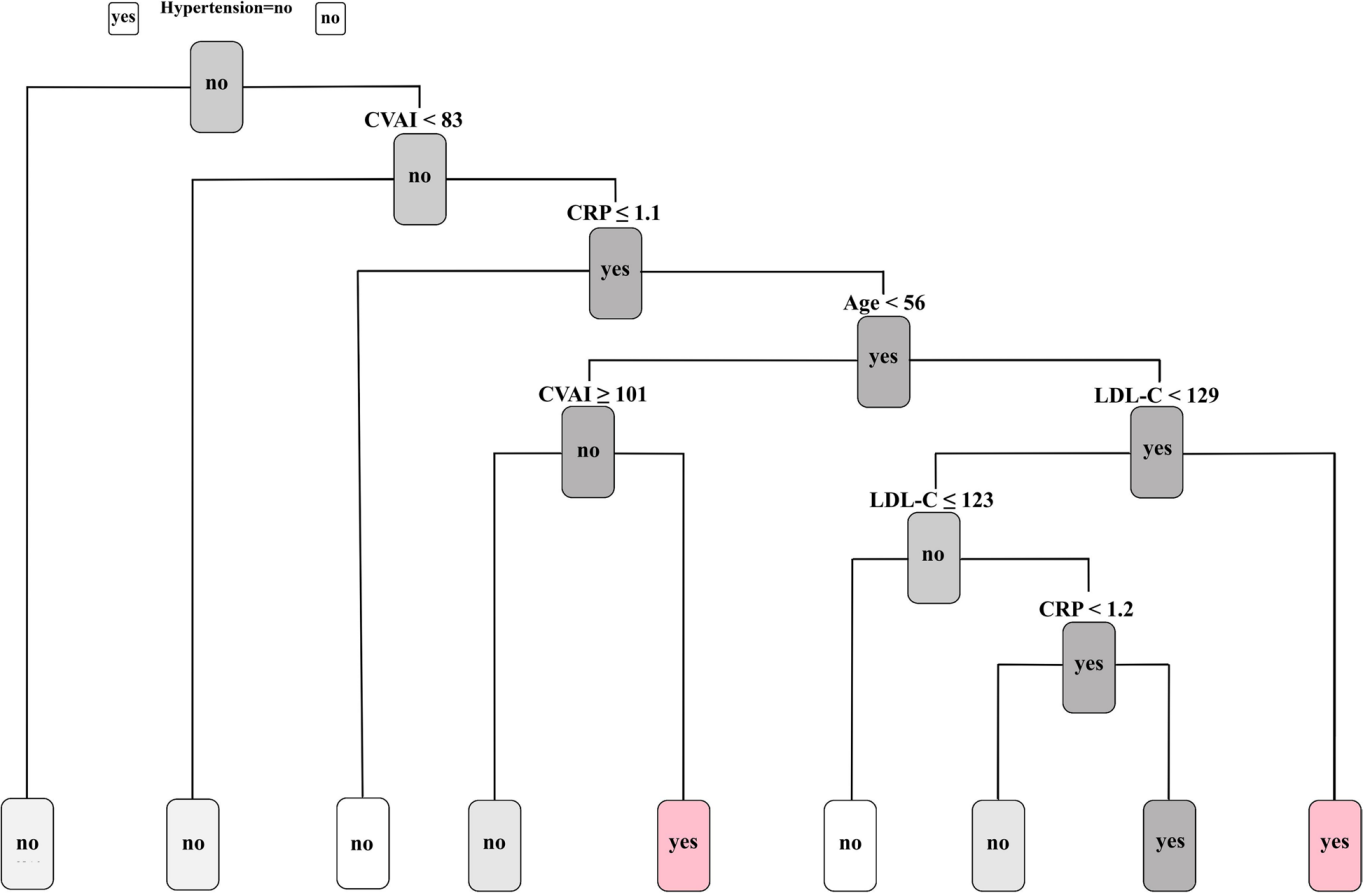
Decision tree analysis integrating demographic and clinicopathological attributes. The illustration indicates that hypertensive patients with CVAI ≥ 83 and CRP ≥ 1.1 mg/l exhibit a predictive value for new-onset stroke events. CVAI, Chinese Visceral Adiposity Index; CRP, C-reactive protein

To appraise the prognostic utility of CVAI, we conducted a decision tree analysis incorporating demographic and clinicopathological features of new-onset stroke occurrences (Fig. 4). The resultant model demonstrated an accuracy of 84%. The analysis revealed a heightened stroke risk in hypertensive individuals presenting with CVAI ≥ 83 and CRP ≥ 1.1 mg/l.

### Stratified analysis

The aforementioned DAG facilitated the identification of pertinent adjustment factors, thereby eradicating confounding variables, which in turn mitigated bias in estimating the association between CVAI and the new-onset stroke in the final model. The considered covariates for adjustment included demographic and behavioral factors such as sex [32], marital status [33], educational attainment [34, 35], geographic location [36], smoking habits [37], and alcohol consumption [38], along with diseases history like presence of diabetes [39], hypertension [40], LDL-C [41, 42], and CRP levels [43].

**Fig. 5:**
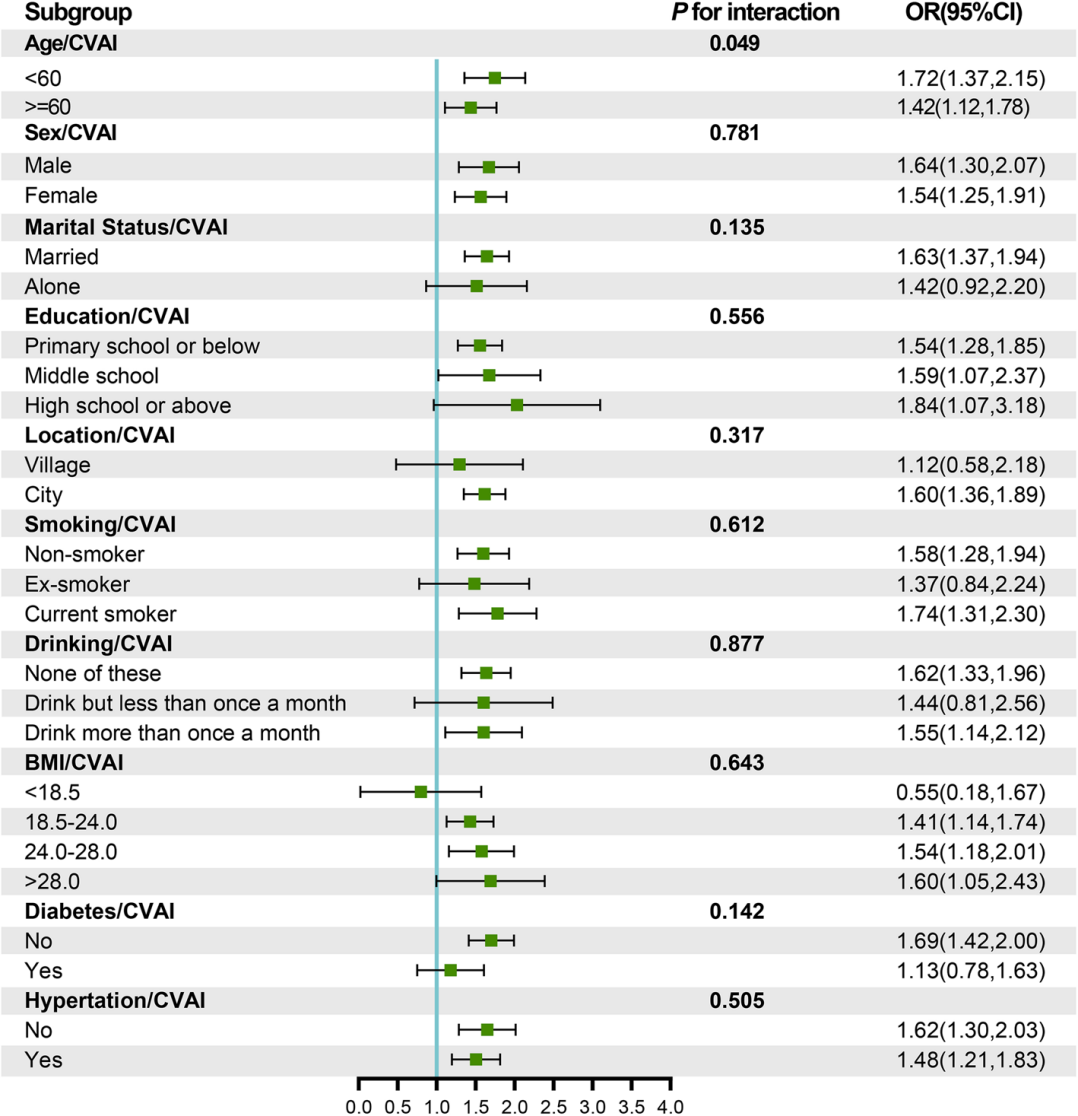
Stratified analysis assessing the interaction effect of CVAI on new-onset stroke incidence across varied subgroups. The forest plot reveals that age may exert moderating influences on the correlation between CVAI and new-onset stroke occurrences (*P* for interaction < 0.05). CVAI, Chinese Visceral Adiposity Index

Figure 5 displays the outcomes of the stratified analysis. Participants were segregated into distinct subgroups based on specific characteristics to ascertain potential variations in the interaction effect of CVAI on new-onset stroke incidence across diverse subpopulations. The findings indicated that age could serve as a moderating factor in the relationship between CVAI and new stroke events (*P* for interaction < 0.05). Notably, among individuals below 60 years of age, each IQR increment in CVAI corresponded to a 72% elevated stroke risk (OR: 1.72, 95% CI: 1.37–2.15). Conversely, participants exceeding 60 years of age exhibited a 42% elevated risk (OR: 1.42, 95% CI: 1.12–1.78).

### Incremental prognostic value of CVAI

Table 3 demonstrates that incorporating CVAI into the fundamental model significantly enhanced the C-statistic for novel stroke incidence prediction. This integration led to substantial improvements in both continuous net reclassification improvement (NRI) and integrated discrimination improvement (IDI), increasing by 0.260 and 0.005, respectively (*P* < 0.001).

**Table 3:** Incremental prognostic value of CVAI

**New-onset Stroke**	**C-statistic Estimate (95% CI)**	***P***	**NRI (continuous) Estimate (95% CI)**	***P***	**IDI Estimate (95% CI)**	***P***
Basic model	0.655 (0.628, 0.681)		ref		ref	
Basic model + CVAI	0.676 (0.650, 0.702)	0.003	0.260 (0.162, 0.358)	< 0.001	0.005 (0.003, 0.007)	< 0.001

The basic model included sex, education level, location, marital status, health behavior, and history of diseases, including smoking status, drinking status, LDL-C, CRP, and history of hypertension and diabetes

## Discussion

This prospective cohort study investigated the association of CVAI and new-onset stroke in middle-aged and older Chinese individuals. Upon adjustment for pertinent confounders, an IQR elevation in CVAI was associated with a 57% augmented risk of incident stroke (OR: 1.57, 95% CI: 1.34–1.83). The dose–response analysis showed a linear association of CVAI and the hazard of new-onset stroke.

This research reveals that CVAI can predict the hazard of new-onset stroke among middle-aged and older Chinese adults. In comparison to other indices (TyG-WC, TyG, LAP, TyG-BMI, and VAI) (*P* < 0.001), CVAI had the largest area under the receiver operating characteristic curve (0.674) and may be the most accurate predictor of new-onset stroke, consistent with the findings of Yang et al. [44]. However, their study population was predominantly rural Chinese, whereas this study population was drawn from a nationally representative cohort comprising 450 villages in 150 districts across 28 provinces. A recent study reported that the incidence of new-onset stroke and related risk factors vary between rural and urban locations in developing countries [45]. A decision tree analysis was also conducted to explore the predictive value of CVAI. A stratified analysis was conducted to determine the interaction effect of CVAI on new-onset stroke in various subgroups.

Smoking is one of the major risk factors for stroke. As shown in Table 1, the ‘sick quitter’ effect [46] might explain the findings, namely, the higher proportion of ex-smokers and lower proportion of current smokers in the stroke patient cohort. The phenomenon known as the ‘sick quitter’ effect [47] suggests that the emergence of health-threatening symptoms in a smoker, prior to the onset of a stroke, can prompt cessation of smoking. Although these participants have ceased smoking, their previous history put them at a higher risk of stroke, thereby the higher proportion of ex-smokers compared to current smokers.

In the adjusted cubic spline model, CVAI was linearly linked to the risk of new-onset stroke (*P* overall < 0.001, *P* nonlinear = 0.562). Visceral obesity is a significant risk factor for new-onset strokes. These findings are consistent with previous studies demonstrating that visceral obesity leads to deep white matter lesions through increased pro-inflammatory cytokines [48]. Higher waist-to-hip ratio (WHR) and BMI result in an elevated deep-to-periventricular white matter hyperintensities ratio through elevated interleukin-6, which can increase the likelihood of stroke occurrence. This dose–response curve also revealed that the hazard of new-onset stroke gradually increased with a constant increase in CVAI, highlighting the importance of maintaining CVAI at a suitable level.

Visceral adipose tissue is essential for energy and nutritional metabolism. A recent Mendelian randomization study [49] provided potential evidence for a causal role of visceral adipose tissue in new-onset stroke. However, it also releases inflammatory cytokines and chemokine mediators that contribute to the formation of carotid plaques and elevate the likelihood of new-onset stroke [50]. According to a study by Bi et al. [51], CVAI demonstrated a positive association with the propensity for carotid plaque development. As components of the CVAI formula, a previous large-scale prospective study discovered that WC was positively linked with the risk of subarachnoid and cerebral hemorrhages. In contrast, BMI exhibited an inverse correlation with the likelihood of ischemic cerebrovascular events and intracranial hemorrhage [52]. The results underscore the significance of accounting for visceral adipose tissue when assessing the risk of incident stroke, emphasizing the intricate interplay among BMI, WC, and diverse stroke classifications.

In the present investigation, the decision tree analysis forecasted that hypertensive individuals exhibiting CVAI values ≥ 83, coupled with CRP concentrations ≥ 1.1 mg/l, possess an elevated probability of experiencing a new-onset stroke. This conclusion is consistent with recent findings in the literature. Hypertension constitutes a considerable risk determinant for the emergence of new-onset stroke, as reported by Bangalore et al. [53]. Hypertension also increases the burden of cardiovascular and cerebrovascular diseases [54] and can lead to severe complications, with new-onset stroke being the most common. The decision tree analysis demonstrated a heightened probability of new-onset stroke among hypertensive individuals with a CVAI equal to or greater than 83, coupled with a CRP level no less than ≥ 1.1 mg/l. This result align with those of prior research [55, 56], reaffirming the significant prognostic value of CRP across a spectrum of diseases. As a crucial marker of inflammation in the body, CRP demonstrates a distinct association with both the incidence and prognosis of stroke [57], thus substantiating its role in the prediction and management of this disease; Consequently, individuals falling within the predicted range should engage in ongoing preventive management to control blood pressure, such as self-measurement, and adopt proactive preventive measures to reduce the likelihood of new-onset stroke.

Furthermore, a stratified analysis was executed to explore the association of CVAI and new-onset stroke across different subgroups. The stratified analysis suggested that age could potentially modulate the relationship between CVAI and new-onset stroke (*P* for interaction < 0.1). Notably, a more pronounced interaction effect of CVAI on new-onset stroke emergence was observed in individuals below 60 years of age. This observation might be attributable to the prevalent incidence of ectopic fat deposits in older participants (> 60 years), which is exacerbated by factors such as positive energy balance, obesity, diabetes, and the reallocation of adipose tissue from subcutaneous to visceral regions [58, 59]. This suggests that the effect of CVAI on new-onset stroke is diminished in patients over 60 years of age, highlighting the significance of age in CVAI equations. Compared to a previous study that examined the association between stroke incidence and VAI, CVAI adds the age indicator to its equation to provide a more comprehensive analysis of visceral adiposity in the Chinese population [19, 20]. According to the latest projections by Yao et al. [60], the burden of stroke in the Chinese population over 65 years will significantly increase by 2050, with a projected increase of 104.7% in incidence, 218.5% in prevalence, and 100% in mortality. With the growing aging population and the apparent increase in the burden of new-onset stroke, adequate attention should be given to healthcare and secondary prevention of new-onset stroke in older adults. In order to effectively reduce the incidence of stroke among individuals over the age of 60, it is crucial to adopt multifaceted measures. These measures encompass lifestyle modifications, regular health surveillance, management of concurrent medical conditions, and potentially, societal health initiatives that address wider determinants of health influencing stroke risk. It is essential to manage factors beyond CVAI to effectively mitigate the rise in stroke incidence attributable to an aging population.

### Comparative analysis with prior investigations and contributions to the extant knowledge base

This research stands in contrast to prior investigations by focusing on the associations between the CVAI and new-onset stroke occurrence in middle-aged and older Chinese adults. While previous studies have examined adiposity indices in relation to stroke risk, the present investigation uniquely emphasizes the relevance of CVAI as a culturally specific and demographically sensitive predictor.

Furthermore, this research paves the way for future inquiries into the utility of CVAI in predicting stroke and other health outcomes in distinct populations. Ultimately, the findings presented here serve to enhance the precision and specificity of stroke risk assessments, taking into account the unique cultural and demographic characteristics of the Chinese population.

### Strengths and limitations

The primary strength of this study lies in its use of the CHARLS database, which provided access to a large sample cohort obtained through multi-stage probability sampling from multiple regions. Additionally, a DAG was implemented to select the minimum sufficient set of confounding variables and identify relevant adjustment factors to reduce bias in the approximated relationship of CVAI and new-onset stroke in the final model. Moreover, the current study provides innovative findings to the extant body of knowledge regarding the correlations between the CVAI and the incidence of novel strokes in middle-aged and elderly Chinese individuals, utilizing an observational research approach. These advancements encompass: a) delineating a linear association between CVAI and the likelihood of new-onset stroke occurrence, b) underscoring the potential utility of CVAI as a prognostic instrument for stroke incidence, and c) laying the groundwork for the creation of sophisticated risk prediction frameworks, consequently augmenting the precision and specificity of stroke risk evaluations in this demographic.

However, some limitations should be considered. First, this study was unable to distinguish between types of stroke, such as hemorrhagic or ischemic stroke. Further research is necessary to investigate the association between CVAI and specific stroke types. Second, some of the information utilized in this study, such as information on new-onset strokes, was obtained through standardized questionnaires, which may be subject to information or memory bias [61]. Third, observational analyses may be subject to potential bias and confounding factors. Thus, the causal association between CVAI and new-onset stroke cannot be determined from this study alone. Further genetic investigations, such as a Mendelian randomization design, are necessary to establish the causal relationship. Lastly, several unmeasured potential confounders, such as individual occupation, family status, and socioeconomic status, may have influenced the relationship between CVAI and new-onset stroke through other interacting factors. Furthermore, in the CHARLS database, stroke patients lacked imaging information at the time of onset, and there were a considerable number of participants with missing CVAI data. Finally, this study utilized baseline data from 2011, disregarding alterations in the Chinese Visceral Adiposity Index (CVAI) between 2011 and 2018 due to the unavailability of necessary parameters to compute the 2018 CVAI in the CHARLS database.

## Conclusions

This study found a linear relationship of CVAI and the risk of new-onset stroke within the middle-aged and senior Chinese demographic, with CVAI supplying a predictive scope for stroke initiation. Moreover, the interaction effect of CVAI on new-onset stroke events was more pronounced in participants under 60 years of age relative to those surpassing 60 years. Consequently, hypertensive individuals in the middle-aged and older categories presenting with elevated CVAI should emphasize the prevention of new-onset stroke.

The clinical relevance of this study lies in its potential to guide healthcare professionals in identifying middle-aged and older Chinese adults at a higher risk of new-onset stroke due to increased CVAI. By understanding this association, clinicians can develop more targeted prevention and intervention strategies, focusing on reducing visceral adiposity to mitigate stroke risk in this population. From a future perspective, these findings can serve as a foundation for the development of more refined risk prediction models, incorporating the CVAI alongside other established risk factors. By enhancing the accuracy and specificity of stroke risk assessments, such models could help optimize resource allocation and tailor preventive measures to those most in need, ultimately improving patient care and reducing the burden of stroke in this population.

## Data Availability

The dataset supporting the conclusions of this article are available in the CHARLS database repository, [at http://charls.pku.edu.cn]. To download the data, approval from the CHARLS team is required.

## Abbreviations

CVAI: Chinese Visceral Adiposity Index
CHARLS: China Health and Retirement Longitudinal Study
Q4: Quartile group
DALY: Disability-Adjusted Life Year
GBD: Global burden of disease
WC: Waist circumference
WHR: Waist-to-hip ratio
BMI: Body mass index
HDL-C: High-density lipoprotein cholesterol
TG: Triglycerides
VAI: Visceral adiposity index
DAG: Directed acyclic graph
CRP: C-reactive protein
LDL-C: Low-density lipoprotein cholesterol
OR: Odds ratio
CI: Confidence interval
RCS: Restricted cubic spline
IQR: Interquartile range
NRI: Net reclassification improvement
IDI: Integrated discrimination improvement
